# Positive Selection in Gene Regulatory Factors Suggests Adaptive Pleiotropic Changes During Human Evolution

**DOI:** 10.3389/fgene.2021.662239

**Published:** 2021-05-17

**Authors:** Vladimir M. Jovanovic, Melanie Sarfert, Carlos S. Reyna-Blanco, Henrike Indrischek, Dulce I. Valdivia, Ekaterina Shelest, Katja Nowick

**Affiliations:** ^1^Human Biology and Primate Evolution, Freie Universität Berlin, Berlin, Germany; ^2^Bioinformatics Solution Center, Freie Universität Berlin, Berlin, Germany; ^3^Department of Biology, University of Fribourg, Fribourg, Switzerland; ^4^Swiss Institute of Bioinformatics, Fribourg, Switzerland; ^5^Max Planck Institute of Molecular Cell Biology and Genetics, Dresden, Germany; ^6^Max Planck Institute for the Physics of Complex Systems, Dresden, Germany; ^7^Center for Systems Biology Dresden, Dresden, Germany; ^8^Evolutionary Genomics Laboratory and Genome Topology and Regulation Laboratory, Genetic Engineering Department, Center for Research and Advanced Studies of the National Polytechnic Institute (CINVESTAV-Irapuato), Irapuato, Mexico; ^9^Centre for Enzyme Innovation, University of Portsmouth, Portsmouth, United Kingdom

**Keywords:** primate, transcription factor, speciation, great apes, archaic humans, gene regulatory evolution, phenotypic evolution, KRAB-ZNF

## Abstract

Gene regulatory factors (GRFs), such as transcription factors, co-factors and histone-modifying enzymes, play many important roles in modifying gene expression in biological processes. They have also been proposed to underlie speciation and adaptation. To investigate potential contributions of GRFs to primate evolution, we analyzed GRF genes in 27 publicly available primate genomes. Genes coding for zinc finger (ZNF) proteins, especially ZNFs with a Krüppel-associated box (KRAB) domain were the most abundant TFs in all genomes. Gene numbers per TF family differed between all species. To detect signs of positive selection in GRF genes we investigated more than 3,000 human GRFs with their more than 70,000 orthologs in 26 non-human primates. We implemented two independent tests for positive selection, the branch-site-model of the PAML suite and aBSREL of the HyPhy suite, focusing on the human and great ape branch. Our workflow included rigorous procedures to reduce the number of false positives: excluding distantly similar orthologs, manual corrections of alignments, and considering only genes and sites detected by both tests for positive selection. Furthermore, we verified the candidate sites for selection by investigating their variation within human and non-human great ape population data. In order to approximately assign a date to positively selected sites in the human lineage, we analyzed archaic human genomes. Our work revealed with high confidence five GRFs that have been positively selected on the human lineage and one GRF that has been positively selected on the great ape lineage. These GRFs are scattered on different chromosomes and have been previously linked to diverse functions. For some of them a role in speciation and/or adaptation can be proposed based on the expression pattern or association with human diseases, but it seems that they all contributed independently to human evolution. Four of the positively selected GRFs are KRAB-ZNF proteins, that induce changes in target genes co-expression and/or through arms race with transposable elements. Since each positively selected GRF contains several sites with evidence for positive selection, we suggest that these GRFs participated pleiotropically to phenotypic adaptations in humans.

## Introduction

Phenotypic differences between individuals and species could be partly explained by the sequence differences in coding parts of genes, and partly by the variation in gene regulatory mechanisms (Lewontin, 1974; Wray, 2007; Wittkopp and Kalay, 2012; Lappalainen et al., 2013; Orgogozo et al., 2015; Perdomo-Sabogal et al., 2016; Anderson et al., 2020). The latter can be caused by changes in the DNA sequence of a regulatory region of a gene that could affect its expression (Siepel and Arbiza, 2014), as well as by changes in the sequence of so-called gene regulatory factors (GRFs) that could affect their target genes (Nowick et al., 2011; Perdomo-Sabogal et al., 2014). GRFs are involved in gene regulation in various ways, such as defining timing and tissue-specificity of a gene’s expression. They include proteins such as transcription factors that bind directly to DNA, cofactors that bind to the transcription factors, histone modifying enzymes, and (long) non-coding RNAs (Latchman, 1997; Zhu et al., 2013; Perdomo-Sabogal et al., 2014; Li et al., 2015; Wingender et al., 2015). GRFs usually display pleiotropic characteristics and regulate more than one gene, hence it has been assumed that their sequence, especially of functional domains, should be subject to long-term constraints and conserved even between species (Wagner and Lynch, 2008; Perdomo-Sabogal et al., 2014; Anderson et al., 2020). However, it has also been suggested that the gene regulatory mechanisms evolve under less selective constraints, compared to their target genes (e.g., Anderson et al., 2020). This led to the description of GRFs as having domain-islands of conservation “in a sea of divergence” (Wagner and Lynch, 2008). Non-deleterious evolutionary changes in GRFs regularly occur both within and outside functionally important regions in homeodomain- and zinc-finger (ZNF) proteins, among other GRF families, exemplifying their role for driving intra- and interspecific morphologic innovations and phenotypic diversity (Wagner and Lynch, 2008; Nowick et al., 2013; Perdomo-Sabogal et al., 2014).

Among the most intriguing questions of phenotypic diversity between species are the striking differences between humans and great apes (Nickel et al., 2008; Varki et al., 2008), but also between great apes and other primates. This particular phenotypic diversity cannot be attributed to the sequence differences alone, but must involve expression changes as well (King and Wilson, 1975; Khaitovich et al., 2005). The genetic dissimilarity between humans and their closest relatives, chimpanzees, has been estimated to be 1.2% in average, with slightly higher dissimilarity in non-coding compared to coding regions (Elango et al., 2006; Kronenberg et al., 2018). Taking non-alignable parts of the genome into account, i.e., insertions, deletion, rearrangements, the difference amounts to 3–4%. In contrast, the dissimilarity between humans and the rhesus macaque, a more distant primate species, was estimated to be substantially higher with 6.46% in average, or up to 9.24% when considering small insertions and deletions (Gibbs et al., 2007; Su et al., 2016). Some of the sequence changes could be the outcome of neutral evolution, whereas others could also be the result of ongoing adaptive interactions among genomes and the environment and hence positive selection (Varki et al., 2008). A paramount example for non-neutral selection in a human GRF has been demonstrated for the FOXP2 gene, where two codons seem to be positively selected in humans in comparison to chimpanzees (Enard et al., 2002). Given the genes’ phenotype association, these selected changes were linked to language skills, one of the most distinctive human capabilities (Fisher, 2019).

Depending on the method used for dating, the human lineage diverged approximately 5.5–11.5 million years ago (Ma) from its closest lineage of chimpanzee and bonobo (Patterson et al., 2006; Langergraber et al., 2012; Amster and Sella, 2016; Besenbacher et al., 2019). The resulting human phenotype has been traditionally seen as driven by ongoing adaptations to local environments and niches (Varki et al., 2008; Lachance and Tishkoff, 2013; Jeong and Di Rienzo, 2014). The identification of genes evolving by positive selection can reveal the route in which organisms adapt to their environment (Casola and Hahn, 2009), and answer some substantially important biological questions (Su et al., 2016), for instance, how a specific phenotype arose. It has been long hypothesized that identifying the genes that have been positively selected along the human lineage, in contrast to neutral and purifying selection in their closest relatives (great apes, primates), could offer insight into the biologically significant genetic changes that distinctly characterize humans (Clark et al., 2003; Mundy and Cook, 2003; Nickel et al., 2008; Daub et al., 2017; Goodwin and de Guzman Strong, 2017). Consequently, many studies to date have tested the human genome for signatures of positive selection using several approaches. Several studies aimed at detecting adaptive changes in protein-coding genes in genome-wide scans of a set of primate species (Nielsen et al., 2005; Voight et al., 2006; Sabeti et al., 2007; Kosiol et al., 2008; Goodwin and de Guzman Strong, 2017), usually without a major overlap of positively selected genes among these analyses. Meanwhile, other studies chose an approach to detect the selection on a polygenic level, within groups of genes unified by the function of encoded proteins (e.g., Nowick et al., 2011; Daub et al., 2013, 2017; Afanasyeva et al., 2018).

From some of the first genome-wide scans of primate genomes for positive selection (Nielsen et al., 2005; Gibbs et al., 2007), it was clear that even in the closest primate lineages the adaptive selection pressures could have undergone different paths and left footprints in different genes. In their comparison of macaque, chimpanzee and human genomes, Gibbs et al. (2007) found that only one human gene and as many as 12 chimpanzee genes were uniquely under positive selection, suggesting a lineage-specific selection. Nevertheless, common selective pressures may create uniform selection patterns across a whole set of species (Schultz and Sackton, 2019), necessitating for broader studies of selection on the branch-level (e.g., Daub et al., 2017).

The aim of our study was to identify genes with signatures of positive selection among the primate GRFs. We specifically focused on two branches in the primate tree, the great apes (Hominidae) and human (Hominina) lineages. Since the power to detect positive selection depends on the number of available sequences (Anisimova et al., 2001; Gayà-Vidal and Albà, 2014), we included all 27 currently available primate genomes to add power to our analysis. There are several lists or databases that compile regulatory factors (e.g., Ravasi et al., 2010; Tripathi et al., 2013; Lambert et al., 2018). For this study we chose 3,344 genes from a published human GRF catalog (Perdomo-Sabogal and Nowick, 2019), which we consider to be the most comprehensive GRF catalog to date. Interestingly, positive selection of some of the GRFs from that catalog has been previously proposed among primate species (for instance, 3 of 36 genes in Nielsen et al., 2005; 35 of 187 genes in Su et al., 2016), albeit with fewer species included in the analyses, and at population level within humans (Perdomo-Sabogal and Nowick, 2019). Positively selected mutations are rarely observed as polymorphic sites (Gayà-Vidal and Albà, 2014). Rather, they should have been rapidly fixed by adaptive selection (Gayà-Vidal and Albà, 2014; Slodkowicz and Goldman, 2020). Interestingly, FOXP2 (mentioned above) was shown not to be recently positively selected on the human lineage after thorough investigation of its variation within modern humans (Atkinson et al., 2018; Fisher, 2019). Therefore, investigating the polymorphism of positively selected codons at population level enables the exclusion of potential false positive candidates identified on the species level.

Here, we compile a high quality set of primate GRFs under positive selection by (1) taking advantage of the completeness of our input data (2) by extensive filtering and curation of the input data to reduce false positives and (3) by verification of potential sites under positive selection by inclusion of chimpanzee and human population variation data.

## Materials and Methods

### Compilation of a Primate GRF Data Set

Starting with the list of Ensembl IDs of human GRFs (Supplementary Data 1 from Perdomo-Sabogal and Nowick, 2019), the orthologous coding sequences from 27 primate genomes, including human, available at Ensembl/Compara (Vilella et al., 2009) and NCBI GenBank were downloaded using *biomaRt* (Durinck et al., 2005) and *rentrez* (Winter, 2017) R packages. Thus all gene sequences were from the Ensembl release 100^1^ (Yates et al., 2020) and NCBI GenBank Release 237^2^, both from April 2020.

The age of the GRF relative to the species tree was taken from GenTree^3^ (Shao et al., 2019). In parallel, genome-wide prediction of transcription factor sequences was made. The protein sequences were downloaded from Ensembl. InterPro (Blum et al., 2021) domain annotations were run for each proteome using Blast2GO (Götz et al., 2008). The resulting genome-wide domain prediction tables were confronted with a manually curated collection of TF-type DNA-binding domains (DBD) using an R script. To be considered as a TF, a protein had to possess at least one TF-type DBD. The TF-type DBD list was collected as described in Shelest (2017). In brief, InterPro database was scanned for DNA binding domains excluding non-TF DBD types (such as, e.g., helicases, nucleases, DNA repair enzymes, etc.). The obtained set was confronted with the DBD list from the DBD database (Wilson et al., 2008), which helped to clean the set from non-TF domains, and then was additionally cleaned from redundancies. Plant-specific DBDs were not included in the final list. The proteins were further arranged in TF family groups as described in Shelest (2017).

Alternative splicing could produce false positive results in the positive selection analysis, if non-orthologous exons were aligned. Therefore, for each gene the human MANE (Matched Annotation between NCBI and EBI) transcript isoform was selected as the representative human sequence, and a temporary dataset was created, which contained that isoform and the sequences of all isoforms of non-human orthologs. These orthologous sequences were then clustered using the *MMseqs2* program (Steinegger and Söding, 2017), with at least 80% identity of sequences within clusters, and a cluster containing the human sequence was selected for further analyses.

The selected sequence cluster was stored in a DNAStringSet and converted into an AAStringSet containing the protein sequences using the translate function from the R package *biostrings* (Pagès et al., 2019). A multiple sequence alignment was then created from the amino acid fasta file by MAFFT (Katoh and Standley, 2013). With the output alignment and the original DNA sequence, the codon alignment was created using the program PAL2NAL (Suyama et al., 2006). The phylogenetic species tree of primates, needed for the analyses, was downloaded from the 10kTrees Project v.3^4^ (Accessed March 1st 2020, Arnold et al., 2010). If the ortholog was not found in all genomes, this species tree was adjusted with the function drop.tip from the R package *ape* (Paradis and Schliep, 2019). Using one universal topology of the species tree made the analyses robust to differences in substitution rates among genes, but also to the fact that gene regulatory factors frequently produces distorted gene trees (Anderson et al., 2020).

### Branch-Site Analysis of Positive Selection

Selective pressure acting on protein-coding genes is regularly quantified by estimating the ratio of non-synonymous to synonymous substitutions (ω) between the coding parts of homologs. We detected branches under positive selection by employing two different maximum likelihood methods: the branch-site model (Yang and Nielsen, 2002) using CODEML of the PAML v4.9 suite (Yang, 2007), and aBSREL (Smith et al., 2015) of the HyPhy v2.5 suite^5^. As applied here, both methods calculate the probability of positive selection (*ω* > 1) of a fraction of sites in a predefined foreground branch of the species tree, namely both the human lineage (taxonomically, subtribe Hominina), and the great apes branch (taxonomically, family Hominidae). The age of divergence of these branches from their sister branches is 5.5–11.5 Ma, 16–26 Ma, respectively (e.g., Dos Reis et al., 2018; Besenbacher et al., 2019).

ABSREL additionally allows for different selection pressures (ω) acting on different branches, while the CODEML branch-site model assumes constant *ω*-values for the respective site classes in all background branches (Yang and Nielsen, 2002; Smith et al., 2015). The human lineage is especially interesting as it could shed more light onto the phenotypic evolution of human species, while the great ape lineage could contribute to our knowledge about the divergence of great apes from other Old World monkeys and gibbons. In both, CODEML and aBSREL methods, the empirical *p*-values were obtained assuming a χ^2^ distribution of the log-ratio tests (LRT). Multiple testing of a large number of GRFs was accounted for by the Benjamini-Hochberg method.

Given that alignments of non-homologous positions are known to frequently cause false positives in such analyses (Fletcher and Yang, 2010), we visually inspected those GRF alignments that showed signs of positive selection. If necessary, the alignments were manually corrected in MEGA X software (Kumar et al., 2018) and the analyses repeated. When the LRT suggested positive selection in the CODEML framework, the Bayes empirical Bayes (BEB, included in PAML v4.9) method was used to calculate posterior probabilities and identify codons that might represent positively selected sites (PSS) in the foreground branch (Yang, 2007). In the same manner, we ran MEME from HyPhy v2.5 (Murrell et al., 2012) in order to detect PSS for genes that were positively selected according to aBSREL. Our further analyses focused on candidate genes and codons, for which positive selection was supported by both methods.

### Further Analysis of Positively Selected GRFs and Sites

In order to comprehensively understand the adaptiveness of positively selected sites, we need to define the impact of the particular codon change on the phenotype. The positively selected gene candidates, retrieved as explained above, were manually investigated by mining several genetic and protein databases. We searched for publications covering functions and associations of positively selected GRFs with human phenotypes and diseases in UniProt^6^ (UniProt Consortium, 2019), Ensembl (see text footnote 1; Yates et al., 2020), NCBI databases (see text footnote 2), OMIM^7^ (Online Mendelian Inheritance in Man, 2020) and FANTOM5/FANTOM CAT^8^ (Hon et al., 2017; Kawaji et al., 2017). In order to uncover common pathways and themes in our set of positively selected gene candidates, we looked at the gene ontology (GO) and KEGG pathway classifications, excluding the gene expression related terms. We used the Expression Atlas^9^ (Papatheodorou et al., 2020), ProteomicsDB^10^ (Samaras et al., 2020), and Bgee^11^ (Bastian et al., 2021) to investigate the candidates’ expression pattern among human and other available primates’ tissues and potential differential expression. Possible interactions and co-expression between the positively selected GRFs were investigated by performing string-based protein-protein interaction network analysis with STRING v11^12^ (Szklarczyk et al., 2019), calculating the proteome co-regulatory network with ProteomeHD (Kustatscher et al., 2019), and by mining the EdgeExpressDB (FANTOM4-EEDB^13^, Kawaji et al., 2009). The position of positively selected codons in relation to protein domains and functional sites was checked at UniProt and manually, following the specific protein-related literature. A short summary on used databases is given in Table 1.

**TABLE 1:** List of mined databases with the description of stored information and covered topic.

**Database**	**Description**
Ensembl	Provides access to genomes, their annotation information, domains, structures, external links and some analysis tools. In addition, it contains information on variation for human and chimpanzee genomes, and population-based distribution of the variation
EMBL-EBI Expression Atlas	Provides the freely available information about gene and protein expression, from microarray, bulk and single cell RNA-Seq studies
NCBI (National Center for Biotechnology Information)	Provides access to gene, genome and protein sequences, structure and annotation information, publications, as well as information on genome variation (for instance, SNPs)
UniProt	Contains various general information on proteins, their sequence and structure, function, domains and ontology
OMIM (Online Mendelian Inheritance in Man)	Contains information on known mendelian disorders and focuses on the relationship between phenotype and genotype
GO (Gene Ontology)	Contains information on the functions of genes, together with their hierarchical classification into functional categories
Kyoto Encyclopedia of Genes and Genomes (KEGG)	Provides information on a large array of high-level functions of genes and proteins, collecting their orthologs, metabolic pathways, disease-related network variation etc.
ProteomicsDB	Provides information on human proteome, isoforms of proteins, expression per tissue, and other analytics
Bgee	Retrieve and compare gene expression patterns between animal species
STRING	Contains information on protein-protein interactions
ProteomeHD	Contains information on co-regulation between the proteins, with additional analytics and GO terms
EdgeExpressDB (FANTOM4-EEDB)	Provides information on co-expression networks between expressed components of mammalian genomes
FANTOM CAT (FANTOM5)	Provides atlases of functional parts of mammalian genomes such as promoters, enhancers, lncRNAs and miRNAs, together with metadata

The variation of the PSS in modern humans was investigated in the Ensembl genome browser. The population data therein include, among others, results from 1,000 Genomes project, NCBI ALFA, Gambian Genome Variation Project, GnomAD, TOPMed, ExAC, and Korea1K project. If the ancestral (pre-selection) codon variant was recorded in these projects, the frequency of polymorphisms among populations, their phenotype correlates and calculation of mutual linkage disequilibrium was further investigated in NCBI dbSNP database and Ensembl. In order to be conservative in detecting PSS, we discarded the PSS that showed polymorphisms in modern human populations. However, that does not fully discard the possibility that these PSS could have been positively selected in some time period, either in recent times (but not reaching fixation yet), or in ancient times (so that nowadays new variants relaxed from the selective pressure appear). The sequence and variation of PSS in archaic humans (Vindija and Altai Neanderthal, Denisovans) was read from the Ancient Genome Browser of the Max Planck Institute for Evolutionary Anthropology, Leipzig^14^. Taking archaic variation into consideration, we could approximately date the positive selection process to before or after separation of Neanderthals and Denisovans from Anatomically Modern Humans. The polymorphism of human PSS in non-human great apes was investigated in data from a number of publicly available and published datasets (Prüfer et al., 2012; Prado-Martinez et al., 2013; Scally et al., 2013; De Manuel et al., 2016; Fontsere et al., 2021), providing information from 111 chimpanzees, 17 bonobos, 42 gorillas and 10 orangutans (Supplementary Table 4). For each gene that includes PSS, we aligned all matching reads from these individuals, which allowed us to infer the state and variation of PSS among great apes.

## Results

Not every human GRF had orthologs in all 26 other primate species’ genomes. One of the reasons is the incompleteness of many genomes under investigation, that limits the possibility of multispecies sequence alignment and comparison (Kronenberg et al., 2018). Furthermore, we excluded read-through transcripts and GRFs originating from recent duplication events in the human lineage, i.e., after Homo-Pan divergence, as no thorough orthology relationship could be established for those cases. Even though it is recommended to include duplicated loci into genome-wide scans for selection (Han et al., 2009), recently duplicated genes often experience gene conversion, which has been shown to elevate false detection of positive selection in paralogs in both site and branch-site models (Casola and Hahn, 2009). Our conservative approach resulted in a set of 3,221 protein-coding genes, with 72,086 non-human orthologs (Supplementary Table 1).

**FIGURE 1:**
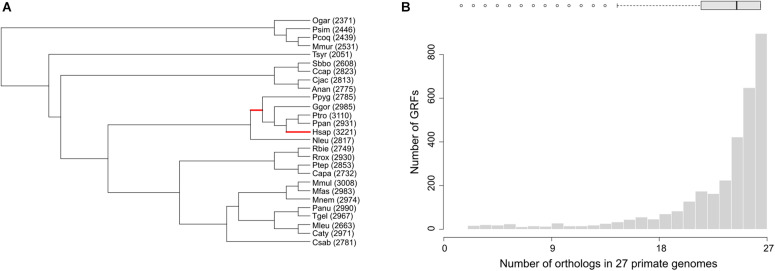
**(A)** Schematic representation of the primate species tree used for the analyses, based on 10 kTrees (Arnold et al., 2010), with analyzed branches (human and great apes) shown in red color. Branch lengths do not represent evolutionary distances. The number of GRFs within specific genomes that was included in the analyses, after filtering the read-throughs and recent duplications, is given in brackets following the species abbreviation. Species abbreviations: Ogar, *Otolemur garnetti*; Psim, *Prolemur simus*; Pcoq, *Propithecus coquereli*; Mmur, *Microcebus murinus*; Tsyr, *Tarsius syrichta*; Sbbo, *Saimiri boliviensis*; Ccap, *Cebus capucinus*; Cjac, *Callithrix jacchus*; Anan, *Aotus nancymaae*; Ppyg, *Pongo abelii*; Ggor, *Gorilla gorilla*; Ptro, *Pan troglodytes*; Ppan, *Pan paniscus*; Hsap, *Homo sapiens*; Nleu, *Nomascus leucogenys*; Rbie, *Rhinopithecus bieti*; Rrox, *Rhinopithecus roxellana*; Ptep, *Piliocolobus tephrosceles*; Capa, *Colobus angolensis palliates*; Mmul, *Macaca mulatta*; Mfas, *Macaca fascicularis*; Mnem, *Macaca nemestrina*; Panu, *Papio anubis*; Tgel, *Theropithecus gelada*; Mleu, *Mandrillus leucophaeus*; Caty, *Cercocebus atys*; Csab, *Chlorocebus sabaeus*. **(B)** Distribution of the number of GRFs having a particular number of orthologs.

The distribution of the total number of detected orthologs in each genome, after applying the filters, as well as the number of GRFs that arose in the specific clades, is shown in Figure 1A. Only 3,044 human GRFs from our dataset were dated by GenTree, and of those, 78 arose within the primate clade. However, 177 studied GRFs were not dated by GenTree, half of them belonging to the zinc finger family previously seen as harboring many primate-specific genes (Nowick and Stubbs, 2010). The median number of orthologs per GRF was 25, meaning that we could identify orthologs in almost all investigated species. Nevertheless, some GRFs have clearly fewer orthologs, either due to their recent origin within primates or due to missing data (Figure 1B).

**FIGURE 2:**
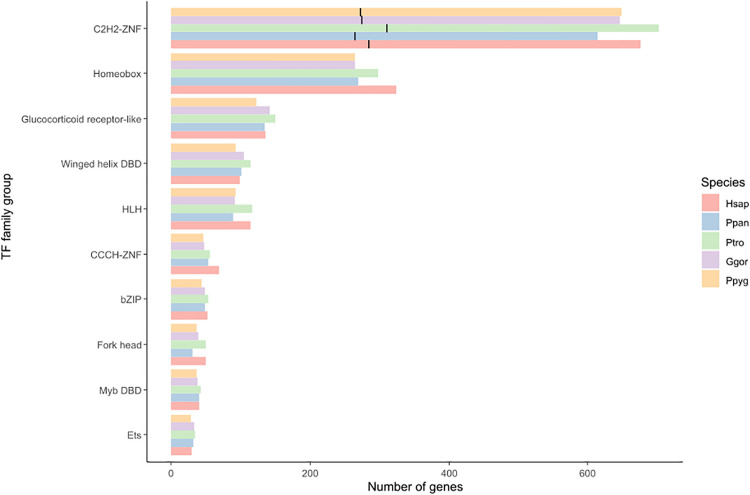
The 10 most abundant families of great apes GRFs. Within the C2H2-ZNF family, the number of KRAB-ZNF genes is marked with a black bar. Species abbreviations are the same as in Figure 1A.

Transcription factors are usually classified into families based on their DNA-binding domain (Wingender et al., 2013; Wingender et al., 2015; Shelest, 2017). The most common GRF family in the analyzed great ape genomes were zinc fingers (especially C2H2-ZNF) and homeobox, followed by glucocorticoid receptors (Figure 2). Although differences in the number of genes per GRF family between species exist, they were not significant (Chi-squared test, *p* = 1; Supplementary Table 2). Within the C2H2-ZNF family, KRAB-ZNF proteins were the most numerous, with 40–42% of all GRFs in great ape species (Figure 2).

### Positive Selection in the Human Lineage

**FIGURE 3:**
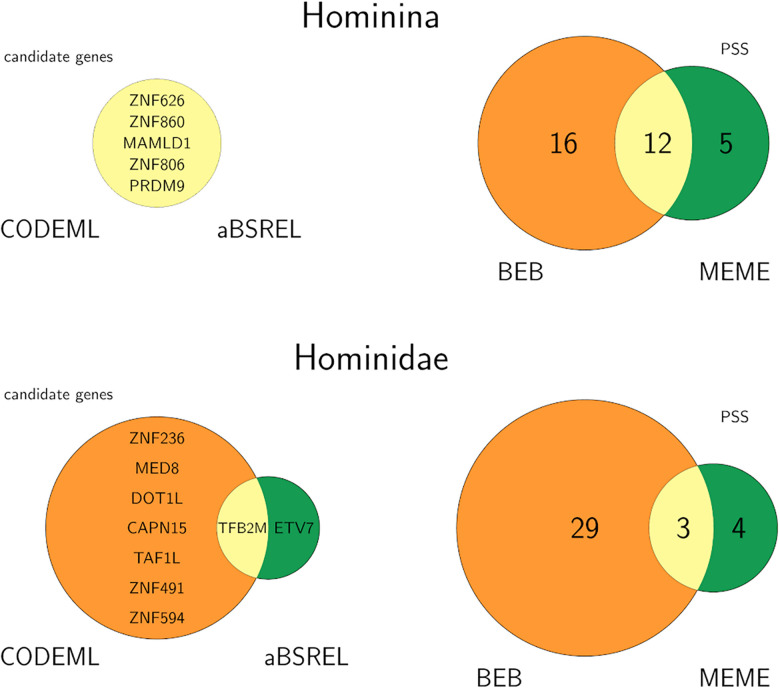
Venn diagrams of PAML (orange) and HyPhy (green) results and their overlap (yellow). The names of the candidate genes and the number of positively selected sites are indicated. Upper row: branch-site selected GRFs (left) and total PSS (right) for the human branch, lower row: the same for the great ape branch.

Since every method is known to produce false positives we decided to perform our analyses with two commonly used packages, PAML and HyPhy, and to keep only the candidates detected by both (Figure 3). CODEML detected 52, and HyPhy 61 candidate genes, before correcting for multiple testing. For the branch-site analysis both procedures indicated the same five genes for positive selection in the human (Hominina) lineage: MAMLD1, and four KRAB-zinc-finger containing proteins (PRDM9, ZNF626, ZNF806, and ZNF860) (Supplementary Table 3). To learn more about these five candidates, we next investigated their evolutionary age and expression patterns. All candidates seem to have arisen at very different time points. According to GenTree, MAMLD1 arose within the land vertebrate clade (Tetrapoda), PRDM9 is seen as common for placental mammals (even though several studies identify it as the earliest in its protein family, being present already in the ancestors of chordates; Birtle and Ponting, 2006; Imbeault et al., 2017; Helleboid et al., 2019), while three of them appeared in primate clade: ZNF626 within Simiiformes, ZNF860 within Catarrhini, and ZNF806 originated within great apes. This indicates that changes in relatively old and relatively young genes show signs of positive selection on the human lineage.

Further, it seems that different tissues could be affected by these changes. MAMLD1, ZNF626, and ZNF860 are ubiquitously expressed in human tissues, while PRDM9 and ZNF806 are predominantly expressed in testes, developing ovaries, and parts of the central nervous system. Interestingly, MAMLD1 is additionally seen as important for the male gonad development (GO:BP term 0008584), and has the highest expression in gonads in comparison to other organs in almost all primates included in the Bgee database. In the FantomCAT both, PRDM9 and MAMLD1, were associated with testes as well, whereas ZNF860 was associated with B-cells and ZNF626 with middle temporal gyrus. A SNP nearby ZNF626 was associated with bipolar disorder. Finally, no co-expression and protein-protein interaction between all the positively selected candidates was found in FANTOM4-EEDB database nor by STRING analysis, and there was not enough expression data for building a proteome co-regulatory network of these genes at ProteomeHD. Taken together, we did not find a common expression pattern nor sufficient data indicating a functional link between the five candidate genes, but it is worth mentioning that at least three of them might have important roles in gonads or the nervous system.

Within the five positively selected genes in the human branch, a total of 33 codons was detected as positively selected sites (PSS) by at least one of the BEB or MEME procedures (Supplementary Table 3). Of those, 12 within three genes were detected by both procedures (Figure 3 and Table 2).

**TABLE 2:** The 11 positively selected codons (PSS) within three genes with positive selection in the human branch that were detected by BEB and MEME, along with the respective nucleotide and amino acid (in brackets) changes.

**Gene/PSS**	**Nucl(AA) change**	**SNP in modern human**	**Decision**
**MAMLD1**			
726	AGT (S) > AGA (R)	/	True PSS
728	GGC (G) > GAC (D)	/	True PSS
**PRDM9**			
155	CCT (P) > TCT (S)	/	True PSS
573	ACA (T) > ATA (I)	rs199686868	True PSS
591	CGG| CAG| GTT (R| Q|V) > TGG (W)	rs200381384	True PSS
629	ACA (T) > AGA (R)	rs112192848	True PSS
657	ACA (T) > AGA (R)	rs112679149	True PSS
681	AG[A| T] (R| S) > ACT (T)	rs6875787	Minor allele
737	TGT| ATT (C| I) > AGA (R)	/	True PSS
**ZNF860**			
219	CAA (Q) > CTA (L)	/	True PSS
348	GAC (D) > GAA (E)	rs13064905	False positive
464	[A| C]GT (S| R) > CAT (H)	rs1808125	False positive

**The existence of non-synonymous single nucleotide variation (SNP) in modern humans is also included, as well as the decision on PSS after applying a rigorous quality check.**

In MAMLD1 there were two candidate PSS detected, that involved an exchange of amino acids with different physico-chemical properties. These codon sequences are fixed without variation in human populations (Table 2). At the same time, they are not variable among bonobo, chimpanzee and gorilla populations (Supplementary Table 4). These features make them ideal candidates for positively selected sites.

PRDM9 exhibits a strong signature of positive selection, which empowers the identification of seven PSS, all of which but one are distributed among six of 14 zinc-finger domains present in the protein. Most changes cause alterations of amino acid properties. The codons 573, 629, and 657 are located between the histidine residues of the zinc finger domain that coordinate the zinc ion, while the codons 591 and 737 are at the α-helix positions –1 and 6, respectively, that specify DNA-binding (Brayer et al., 2008; Oliver et al., 2009). These positions were also among the three positions found to be under positive selection by Oliver et al. (2009). PSS in PRDM9 show variation within humans, with occasional appearance of the ancestral state, i.e., the sequence seen in great ape genomes (Table 2). These mutations are rare in most human population samples, reaching for the closely positioned SNPs rs199686868 and rs200381384 (codons 573 and 591) frequencies of 0.02 and 0.06 in Gambian and Korean populations, respectively. Even though positioned in codons that are close to one another in the genome, these SNPs do not exhibit linkage disequilibrium in the Gambian population (Ensembl) and can therefore be seen as independent and furthermore, not a result of a recent selective sweep. Codons 629 and 657 represent the same substitution, at the same position in relation to functional histidines of two neighboring ZNF domains. This is a result of already recognized concerted evolution within PRDM9 (Oliver et al., 2009; Schwartz et al., 2014). The population-wide alignment of Pan PRDM9 sequences showed high diversity in the number of ZNF domains, but also in their sequence at the DNA-binding sites (Groeneveld et al., 2012). The homologous DNA-binding position (–1) in the fourth ZNF domain of any Pan sequence does not harbor the human-specific codon 591 (TGG). The homologous position (+6) of codon 737 is seen in two recognized PRDM9 zinc finger alleles in Pan (alleles D and Z in Groeneveld et al., 2012), with one other DNA-binding position (+3) of these alleles being the same as in humans. Population stratification of both Homo and Pan PRDM9 sequences was previously shown by Schwartz et al. (2014).

**FIGURE 4:**
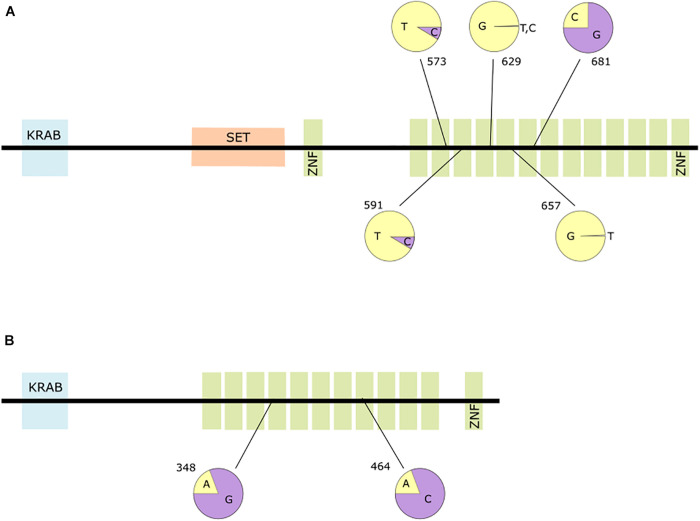
Domain structures of **(A)** PRDM9; **(B)** ZNF860. Green boxes indicate ZNF domains. The pie charts represent frequencies of the positively selected (yellow) and ancestral variants (purple) for the respective positions within the proteins.

Codon 681 of PRDM9 constitutes an exception of this set of PSS as the ancestral codon state (AGT) is described as rs6875787. Not reported in the 1,000 Human Genomes Project, this polymorphism was nonetheless found in other population genomics projects (for instance, in GnomAD genomes dataset), with frequencies of the “ancestral” nucleotide G of around 0.75 in the overall modern human population (Figure 4A), and “positively selected” C of around 0.25. Moreover, we cannot conclude about the presence of this polymorphism in Neanderthal populations, due to limited data. Most of the chimpanzee and bonobo ZNF alleles of PRDM9 have AGT at the DNA-binding position 6 (Groeneveld et al., 2012). This PSS candidate constitutes rather a case of human genetic variation where one of two major alleles was chosen to be the reference. It may also be that this region is located within a region biased for the sequencing technology used in some genome projects leading to an omission of that SNP. In any case, we rule it out as a true positively selected site in the human lineage.

Two of three PSS candidates in ZNF860 (codons 348 and 464) lay within the zinc finger domains. Their ancestral codon states, as well as the ancestral codon states of the candidate obtained by MEME only (codon 609), are reaching the frequencies up to 0.80 and 0.90 in African populations (Figure 4B). They more likely represent the ancient variations within the human lineage, than reverse mutations to the ancestral states. The archaic humans have either ancestral, modern or heterozygous states of these codons. These three codons also stand in linkage disequilibrium, with correlation (r^2^) between alleles 0.5–1. This implies that the alleles we got as positively selected are part of one haplo-block. We regard these codons as false positives, induced by variation that is not present in the human reference genome, as well as a potential result of bottleneck in non-African human populations. This leaves ZNF860 with only one PSS, codon 219. This codon shows no variation within the analyzed bonobo, chimpanzee, gorilla and orangutan samples (Supplementary Table 4).

In total, our strict analysis recovered nine codons that show signs of positive selection. All nine PSS had the same sequence in the archaic humans (Neanderthals and Denisovans). Even with the caveat of limited data for these genomes, there was no polymorphism detected at the PSS in the high coverage sequenced archaic humans. It can be concluded that the adaptive selection happened along the human branch, before the divergence between Neanderthals, Denisovans and anatomically modern humans.

In ZNF626, seven sites were detected to be positively selected by the BEB method only. Interestingly, three of them are located within the KRAB domain of the protein. Additionally, a frameshift mutation occurred in the human gene within codon position 503, degenerating its last, 12th zinc finger domain. Fourteen codons after the frameshift were detected by both MEME and BEB, and were thereafter excluded as false positives.

We found that ZNF806 is part of a series of duplications within great apes. First, ZNF285 was duplicated in apes branch (Hominoidea) giving rise to ZNF285B, that is positioned nearby, but on the other strand of chromosome 19. The high similarity of those recently formed paralogs can lead to genome assembly mistakes as seen in our analysis for the fragmented gibbon genome. For this reason, gibbon ZNF285 and ZNF285B had to be excluded from our respective analyses. The next duplication yielded ZNF806 in all great ape genomes, positioned at human chromosome 2 or its homolog 2B, or on an unplaced scaffold in the orang-utan genome. Yet another duplication happened in the Homo/Pan branch yielding a paralog, present in all three available genomes at chromosome 20. Although all four paralogous genes have similar sequences, their orthology relationship was not resolved in Ensembl/Compara. The gene tree built in MEGA X (Kumar et al., 2018) supports a duplication of ZNF806.

### Positive Selection in Great Apes

In the great ape lineage, CODEML and aBSREL gave different results, overlapping only in one gene (Figure 3). Positive selection was detected by CODEML in eight GRFs (CAPN15, DOT1L, MED8, TAF1L, TFB2M, ZNF236, ZNF491, ZNF594), and by aBSREL in two (ETV7, TFB2M). The single overlap was gene TFB2M, which is expressed in all human tissues. TFB2M is a nuclear gene that is a part of the mitochondrial transcription initiation complex, and as such it is required for basal transcription of mitochondrial DNA (Falkenberg et al., 2002) but also for replication and packaging of mtDNA and ribosome biogenesis (Bonawitz et al., 2006). This gene is seen as having a critical role in mitochondrial DNA gene expression, and mutations in the gene or deviation in its expression have been associated with mitochondrial DNA depletion syndromes, Parkinson disease (Grünewald et al., 2016), and autism spectrum disorder (Park et al., 2018). Within TFB2M, both BEB and MEME methods detected three PSS that are not located within any domain. None of these PSS were variable within human and non-human population data we analyzed.

As the overlap between the methods was too small to investigate the potential coexpression and interaction between the positively selected genes, we included all the positively selected candidates in the great apes branch in these analyses. However, similar to the positively selected GRFs in the human lineage, there was no co-expression, protein-protein interaction, and co-regulatory network of these genes found by STRING and ProteomeHD.

## Discussion

In our study, starting with a list of over 3,000 human GRFs, we identified five and one orthologs with significant signs of positive selection in the human and great apes lineages, respectively. To our knowledge, three of the identified GRFs, namely ZNF626, ZNF806, and ZNF860, have not been reported in previous analyses of positive selection among primate species (for instance in Nielsen et al., 2005; Su et al., 2016; Van Der Lee et al., 2017). The number of our candidate genes is small in contrast to the genome-wide studies (e.g., Su et al., 2016; Van Der Lee et al., 2017), but is in line with studies that estimated the proportion of adaptive amino acid substitutions as low in humans (Fay et al., 2001; Zhang and Li, 2005; Boyko et al., 2008). Furthermore, we focused only on GRFs, which constitute about one sixth of all protein-coding sequences (20,448 in human reference genome, assembly GRCh38.p13, Ensembl). We included only 78 of 254 primate specific GRFs (Shao et al., 2019), as the others were found in <3 genomes. We took effort to reduce the number of false positives that likely falsely increased the number of positively selected genes in previous studies. Firstly, in comparison to previous analyses of positive selection within primates, we included more genomes—all the 27 available from Ensembl—thus substantially increasing the power to detect signatures of positive selection. Secondly, we excluded very divergent sequences from our sets of orthologs by performing an ortholog clustering step. High divergence might lead to the saturation of non-synonymous changes and thus affect the power of our branch-site tests (Gharib and Robinson-Rechavi, 2013). Our approach is, however, limited by the incompleteness of non-human primate genomes and the GRF sequences therein. Third, we manually investigated alignments that resulted in detection of positive selection to exclude those cases, where the respective signal was caused by alignment of non-homologous codons. In most cases, improving the alignments resulted in losing the statistical significance. Fourth, we consider only those candidates as reliable, which were detected by both, CODEML and aBSREL, methods. This certainly helped to deflate the false positive rate for detection of selected GRFs in the human lineage, in scope of the recent finding that around 35% of the human genome is subject to incomplete lineage sorting among the African apes (Kronenberg et al., 2018).

Our findings highlight the role of population data leveraged to the detection of signs of positive selection. Given only reference genomes, we detected 12 codons within five positively selected genes in the human lineage. However, looking into modern human population variance, we have found the PSS sequence variants that are the same as seen in the ancestral lineages (present in at least some of the great ape genomes). Some of them were common polymorphisms, some had very low frequencies. The first are either balanced polymorphisms or the result of a selective sweep within distinct human populations. They are, however, not fixed in the human genome, and were discarded as false positives. The low frequency of the latter polymorphisms allowed us to keep them as good candidates. Together with the PSS that showed no polymorphism, they comprise a set of nine positively selected codons in three genes (MAMLD1, PRDM9, and ZNF860). All of them were present in Neanderthal and Denisovan genomes, thus dating the adaptive selection episode(s) to after the divergence of the Pan/Homo branches (∼6.5–7.5 Ma; Amster and Sella, 2016), and before the divergence among lineages that led to Neanderthal, Denisovan and anatomically modern humans 765,000–550,000 years ago.

In order to clearly identify sequence changes that could distinguish human and primate adaptive phenotypes, genetic variation and population data also need to be analyzed from non-human primate species. As seen in one of our positively selected genes, PRDM9, the variation among chimpanzees and bonobos is high (Groeneveld et al., 2012), and there are alleles/haplotypes that are the same as in the human genome. Based on our results, balanced polymorphisms in the human genome, like the ones seen in ZNF860 and PRDM9, as well as polymorphism resulting from incomplete lineage sorting and present in some populations of great apes, are prone to be wrongly indicated as PSS. Those cases remain a methodological challenge for the detection of adaptive selection from (only) reference genomes. We thus investigated variation of the detected PSS in 180 non-human great ape individuals. Three PSS detected for the human lineage showed no variation in either human, or any great ape populations (Table 2 and Supplementary Table 4), strongly indicating that they do not represent incomplete lineage sorting among great apes, but are rather cases of true positive selection. Similarly, the candidate PSS for the great ape lineage within TFB2B are monomorphic, and therefore most likely the true positively selected sites. All in all, our strict approach and inclusion of variation data from great ape individuals led us to obtaining a short but high confidence list of GRFs with signs of positive selection on the human or great ape lineage, and PSS within them.

The adaptive importance of candidate genes should also be seen in their function and interconnections. However, our analysis yielded a small number of positively selected genes. Only one gene, TFB2M, was recovered as positively selected in the great apes branch and having sites under positive selection. This gene is crucial for proper functions of mitochondria as part of the mitochondrial transcription initiation complex that is necessary for expression of all genes encoded in the mitochondrial genome. Mitochondria and mitochondrially-encoded genes are essential for providing energy for all cellular functions. Impaired mitochondria have been associated with several diseases, including mitochondrial DNA depletion syndromes (Correia et al., 2011), diabetes, Parkinson’s, deafness and cancer (Wallace, 2005). The effect of a particular SNP within this gene, c.790C > T, has been seen as delaying the unloading of DNA from TFB2M, thus increasing the mitochondrial DNA expression (Park et al., 2018). If the change in the ease of DNA-TFB2M detachment was also influenced by the PSS we detected, we could speculate at this point, that it might be related to the expression level of respiratory chain complex genes, and may have allowed better energy production efficiency for tissues including the brain.

In the human lineage we detected five positively selected GRFs. Four of them belong to the GRFs that possess a KRAB domain together with zinc finger domains, so called KRAB-ZNF proteins. KRAB-ZNFs themselves constitute the largest class of GRFs within the human genome (Mark et al., 1999; Yang et al., 2017). It is worth mentioning, that even though KRAB-ZNFs represent ∼40% of the C2H2-ZNF protein family in great apes, they constitute 80% of positively selected GRFs in the human lineage in this study. Our results are thus in agreement with earlier findings that KRAB-ZNF proteins evolve rapidly (Nowick and Stubbs, 2010; Nowick et al., 2011; Zhao and Kishino, 2020). Some additional and previously known modes of their evolution, such as changes of zinc-finger copy numbers and loss of KRAB domains (Nowick and Stubbs, 2010; Nowick et al., 2011; Shao et al., 2019) were not within the scope of our analysis, but could also have happened by natural selection.

KRAB-ZNF proteins have been implicated in many important functions, such as genomic imprinting, cell differentiation, metabolic control, brain development, but also phenomena like sexual dimorphism and speciation (Nowick et al., 2013; Jacobs et al., 2014; Yang et al., 2017). Recently, it was discovered that at least some KRAB-ZNFs, such as ZNF91/93, are important for recognition and transcriptional silencing of transposable elements (Jacobs et al., 2014; Helleboid et al., 2019). The KRAB-ZNF family is also enriched among genes with differential expression between human and chimpanzee prefrontal cortex (Nowick et al., 2009).

Three of the KRAB-ZNFs identified in this study (ZNF626, ZNF806, and ZNF860) are largely unexplored to date. ZNF626 was pointed out as a candidate gene involved in posttraumatic stress disorder in European American individuals of the United States Army (Stein et al., 2016) and seems to be associated with bipolar disorder (Hon et al., 2017). It is highly expressed in the middle temporal gyrus, which is involved in language processing, for instance while reading (Acheson and Hagoort, 2013). Hippocampus-specific somatic mutations within ZNF806 have been identified in 9 out of 17 patients with sporadic Alzheimer’s disease (Parcerisas et al., 2014). Previous association of the ZNF806 SNP rs4953961 with tardive dystonia, one of the serious types of extrapyramidal symptoms that antipsychotics can cause, was shown to be erroneous and probably relatable to similar genomic regions (Kanahara et al., 2021). In our study, we have revealed that ZNF806 is in a group with three paralogous ZNF sequences, one of which can be a potential candidate for this symptom. ZNF860 has been associated with early-onset type 2 diabetes mellitus and prostate cancer, and its higher expression is seen as an indicator for gastric cancer (Dmitriev et al., 2015; Yamada et al., 2018; Pan et al., 2019). These findings indicate that these positively selected ZNF genes are playing a role in complex phenotypes. Interestingly, two of those genes, ZNF626 and ZNF806, may be associated with the brain.

PRDM9, on the other hand, is a well-studied gene. It specifies the sites of meiotic DNA double-strand breaks that initiate meiotic recombination in mice and humans. PRDM9 is known to bind to specific DNA sequences with its DNA binding domain, to induce methylation to adjacent nucleosomes, and to recruit or activate the meiotic machinery (Baudat et al., 2010; Billings et al., 2013). Although its function can be seen as essential, a human adult knock-out was reported, pointing to differences in humans vs. non-primate mammals, and supporting the possibility of alternative mechanisms of localizing human meiotic crossover (Narasimhan et al., 2016). PRDM9 was previously reported as a candidate for positive selection in a number of studies (e.g., Oliver et al., 2009; Schwartz et al., 2014; Daub et al., 2015). At least in mice, this gene has been considered as a speciation gene causing infertility in hybrids (Mihola et al., 2009), and a similar role has been proposed for the primate clade (Daub et al., 2015). In addition to that, Schwartz et al. (2014) have speculated that positive selection at positions dedicated to DNA binding and specificity can lead to differential usage of binding motifs, which may result in abovementioned hybrid sterility and contribute to speciation in the primate lineage. Our work further supports a major role for PRDM9 in speciation of humans.

Our fifth candidate, MAMLD1, seems to be important for sex determination and the development of male genitalia. Mutations in MAMLD1 have been found to cause hypospadias type 2, a disorder of sex development in which the male urethral opening is moved ventrally, and genitalia of XY individuals can appear female-like (Fukami et al., 2006). Changing the position of urethral opening will also functionally block successful mating, in terms of delivering sperm into the genital tract of females. Indeed, strong differences in size and morphology of testicles and penis exist between humans and chimpanzees and seem to be related to their mating strategies (Harcourt et al., 1981; Brindle and Opie, 2016). Genes involved in reproduction are considered prime candidates for driving speciation. Several other studies of positive selection in the human genome have also disclosed genes involved in spermatogenesis and transcriptional regulation (e.g., Clark et al., 2003; Gayà-Vidal and Albà, 2014). While PRDM9 might create a species barrier at the postzygotic level, MAMLD1 might have been involved in establishing a barrier prior to fertilization.

However, no co-expression, interaction or co-regulation among our candidate genes was previously reported. It may be speculated that the sets of genes they regulate are independent, or acting in different pathways, such that the epistasis among them could not be detected with the currently available data. The seeming independence and the possibility of participation in complex phenotypes can be accounted for if we include the potential pleiotropic effect. Namely, since GRFs usually regulate the expression of several to many genes, they can induce various physiological and morphological consequences within cells, tissues or at the level of whole organisms (Stern, 2000; Wagner and Lynch, 2008; Wagner and Zhang, 2011). These consequences could be independently adaptive. It has been shown that small mutations, even within a single gene, may provide a rapid path to phenotypic adaptation (Linnen et al., 2013). The different PSS that we identified within one gene, can of course add to the pleiotropic effect and be associated with different traits. This has been reported before for some genes, where different polymorphic sites had different trait associations (Flint and Mackay, 2009; Mackay et al., 2009). Yet another plausible explanation for the lack of interaction among our candidate genes is that the selective pressures on them were acting at different timepoints after the split of the human lineage. There might have been millions of years between the selective events, so that they can likely be considered to have occurred independently from each other.

Taken together, our study points out six candidate GRFs that experienced positive selection in great apes and human branches. These GRFs did not show common patterns of co-expression or co-regulation. Hence, we concluded that the effect of several PSS within some of these genes, could have had pleiotropic effects on different phenotypic traits, and that the effect of all candidate GRFs may have been epistatic toward the same goal—adaptation. Detection of mainly KRAB-ZNF genes as positively selected GRFs in the human lineage, along with the recent duplication events for at least one of them (ZNF806), lead us to propose that these proteins are driving human-specific phenotypes by shifting target genes co-expression (as proposed by Nowick et al., 2009), and through arms race with transposable elements (Imbeault et al., 2017; Yang et al., 2017; Warren et al., 2020). The association with the brain for at least some of them further supports the notion that phenotypic and cognitive differences in the primate brain might have been caused by adaptive changes in regulatory factors.

## Data Availability Statement

The original contributions presented in the study are included in the article/Supplementary Material, further inquiries can be directed to the corresponding author/s.

## Author Contributions

KN, HI, CR-B, and VJ conceptualized and developed the research idea and designed the study. CR-B, ES, DV, MS, and VJ built code pipelines and analyzed the data. VJ, MS, and KN interpreted the obtained results and prepared the manuscript. All authors have read, discussed and approved the final submitted manuscript.

## Conflict of Interest

The authors declare that the research was conducted in the absence of any commercial or financial relationships that could be construed as a potential conflict of interest.

## Footnotes

1 www.ensembl.org/

2 www.ncbi.nlm.nih.gov/search/

3 gentree.ioz.ac.cn

4 https://10ktrees.nunn-lab.org/Primates/

5 www.hyphy.org

6 www.uniprot.org/

7 https://www.omim.org

8 https://fantom.gsc.riken.jp/cat/v1/

9 www.ebi.ac.uk/gxa/home

10 www.proteomicsdb.org

11 https://bgee.org/

12 www.string-db.org/

13 https://fantom.gsc.riken.jp/4/edgeexpress/subnet/

14 bioinf.eva.mpg.de/jbrowse/
